# cophesim: a comprehensive phenotype simulator for testing novel association methods

**DOI:** 10.12688/f1000research.11968.1

**Published:** 2017-08-01

**Authors:** Ilya Y. Zhbannikov, Konstantin G. Arbeev, Anatoliy I. Yashin

**Affiliations:** 1 Biodemography of Aging Research Unit (BARU) at Social Sciences Research Institute (SSRI), Duke University, Durham, NC, 27705, USA

**Keywords:** Phenotype simulation, GWAS

## Abstract

Simulation is important in evaluating novel methods when input data is not easily obtainable or specific assumptions are needed. We present *cophesim*, a software to add the phenotype to generated genotype data prepared with a genetic simulator. The output of *cophesim* can be used as a direct input for different genome wide association study tools. *cophesim *is available from https://bitbucket.org/izhbannikov/cophesim.

## Introduction

Genome-wide association studies (GWAS) are routine in population research. New methods are being developed for better accessing complex associations between genotypes and phenotypes, uncovering genotype structures or testing evolutionary hypotheses. Testing the novel methods requires experimental data, which may not be easily obtainable. One solution is to use artificial data simulated with specific assumptions.

The best existing phenotype simulators, such as: *GENOME* ^1^, *Plink* ^2^, *phenosim* ^3^, *CoaSim* ^4^, *Fregene* ^5^, *ForSim* ^6^, *QuantiNemo* ^7^, *GCTA* ^8^, *HapGen* ^9^, *SeqSimla* ^10^, and *SimRare* ^11^ offer qualitative and dichotomous simulated phenotype. But the known phenotype simulation software tools have some limitations, which may prevent customers from using them: (i) the majority, if not all, of the phenotype simulation software tools do not offer simulation of survival traits/time-to-event outcome, making it impossible to test respective hypotheses of associations; (ii) some of the tools are not easy to use, due to wide range of parameters, which the user has to provide and control (rather than calculate them automatically), making them unnecessarily difficult to use and preventing the user from future use of the tool; (iii) phenotype simulation is often offered as an auxiliary part of the genetic simulation routine, and therefore the user first has to perform a time-consuming unavoidable genetic simulation in order to obtain the phenotype; (iv) in situations when the genetic data is already simulated from other tools, only *phenosim* and *GCTA* offer adding simulated phenotype to such data. Consequently, it is necessary to have a new, simple and flexible phenotype simulation tool with plain algorithmic assumptions.

Consequently, we present *cophesim*, a comprehensive phenotype simulation tool that was developed to add a phenotype to corresponding genotypes simulated by other simulation tool ( Table S1). *cophesim* offers simulation of continuous, dichotomous and survival traits, with different (user-provided) effect sizes of causal variants, with the ability to simulate epistatic interactions. It also can simulate phenotype within gene-environment interaction assumptions using up to 10 covariates.

## Methods

### Implementation

**Figure 1.**
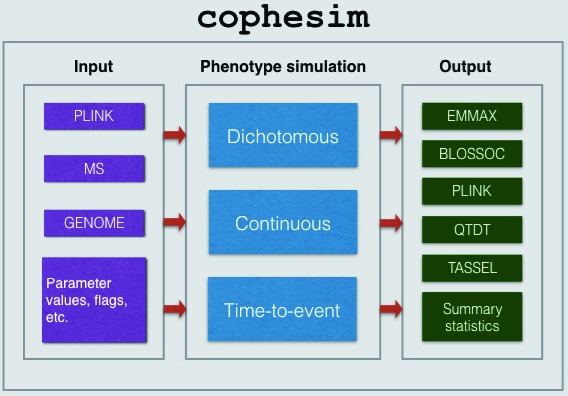
Workflow of *cophesim* has three stages: (1) Input stage, where the input data (can be provided in one of the three formats: *Plink*, *MS* and *GENOME*, see the user manual - Supplementary File 1) along with the other input parameters (such as causal variants with size effects, output format, etc.) is prepared for phenotype simulation; (2) Phenotype simulation stage, where different types of phenotypic traits are simulated: dichotomous, continuous and time-to-event (‘survival’); (3) Output stage – the final stage, where simulated phenotype data are packed to various formats in order to be directly usable by six GWAS tools: *EMMAX*, *BLOSSOC*, *Plink*, *QTDT*, *TASSEL* and *GenABEL*. Summary statistics are generated at the output stage as well.

The workflow (see Figure 1) includes the following stages: (i) Input data pre-processing; (ii) phenotype simulation; (iii) generation of final output files.

### Input data

Currently *cophesim* accepts the genotype output data from *Plink*, *MS* ^12^ and *GENOME* software applications. Phenotypes (dichotomous, continuous and survival) are then added according to the following simulation scenarios.

### Dichotomous phenotype

Dichotomous phenotype for *i ^th^* individual ( *i* = 1... *N*, where *N* is the total number of individuals in a dataset) is simulated according to the logistic model (if the user provided effect sizes for causal variants):

$$p_{i}=\frac{1}{1+e^{-z_{i}}}\;(1)$$

where *p _i_* is the probability of a particular outcome. In *cophesim*, it is a probability of a “case” (cases are marked by “1”, and “0” are controls in simulated dichotomous phenotype) for *i ^th^* individual. If *p _i_* is greater than the some threshold *p*_0_ (we use *p*_0_ ~ *U*(0, 1)), then the phenotype for *i ^th^* individual is set to “1” and to “0” otherwise. The variable **z** is determined with the following equation:

$$z_{i}=\sum_{j=1}^{M}E_{j}g_{ij}+\sum_{j=1}^{K}\alpha_{j}X_{ij}+\epsilon_{i}\;(2)$$

*E _j_* – effect size for *j ^th^* variant, user-defined; *g _ij_* – value of *j ^th^* genetic marker for *i ^th^* individual; *α _j_* - effect size for *j ^th^* covariate and *X _ij_* is a value of *j ^th^* covariate for a *i ^th^* individual (the term $\sum_{j=1}^{K}\alpha_{j}X_{ij}$ is added to represent gene-environment iterations); *ϵ _i_* – a standard normal residual, *ϵ _i_ N* (0, 1), computed for *i ^th^* individual, *M* is a total number of genetic variants and *K* is a total number of covariates used.

If the user did not provide the effect sizes for causal variants, the following strategy is then used:

$$z_{i}=\sum_{j=1}^{M}w_{ij}+\sum_{j=1}^{K}\alpha_{j}X_{ij}+\epsilon_{i}\;(3)$$

Here *w _ij_* is a weight and computed as follows: $w_{ij}=\frac{g_{ij}-2MAF_{j}}{{\left(2MAF_{j}(1-MAF_{j})\right)}^{1/2}}$ (a standardization procedure, and the matrix *W* containing element *w _ij_* is called a standardized genotype matrix ^8^; *MAF _j_* – a minor allele frequency for *j ^th^* genetic variant, and the other values are the same as described above. This strategy allows using defined genetic architecture in a simulated population.

### Continuous phenotype

Qualitative (continuous) phenotype for *i ^th^* individual is simulated according to the linear regression scenario according to the equations (2) or (3) (in case if effect sizes were not supplied).

### Inverse Probability method

We model a survival phenotype from the proportional hazards model using the inverse probability method ^13^: if *U* is uniform in (0, 1) and if *S*(·| **z**) is the conditional survival function derived from the proportional hazards model: *S*( *t*| **z**) = *e* ^– *H*_0_( *t*) *e*^**z**^^, then the random variable

$$T=S^{-1}(\cdot |\text{z})=H_{0}{\left(t\right)}^{-1}\left(\frac{-\text{log}(U)}{e^{z}}\right)\;(4)$$

has survival function *S*(·| **z**). In this equation, *H*_0_( *t*) is a cumulative baseline hazard. By default, we use the Weibull cumulative baseline hazard: *H*_0_( *t*) = *λρt* ^*ρ*–1^; **z** is the same parameter that defined above, for each individual, and depends on whether the user provided effect sizes for causal variants or not. We also implemented exponential and Gompertz hazards.

### Linkage Disequilibrium

The simplest way to simulate collinearity between two SNPs, *g*_1_ and *g*_2_, with effect sizes *E*_1_ and *E*_2_ is to replace some portion of *g*_2_ with *g*_1_ values according to provided $r_{12}^{2}$ coefficient, which reflects a correlation between two SNPs. We also consider applying other techniques, such as copulas, in order to simulate LD.

### Epistatic interactions

These are modeled with the following equation for *i ^th^* individual:

$$z_{i}=E_{1}g_{1i}+E_{2}g_{2i}+E_{12}g_{1i}g_{2i}+\sum_{j=1}^{k}\alpha_{i}X_{ji}+\epsilon_{i}\;(5)$$

where the term *E*_12_ *g*_1 *i*_ *g*_2 *i*_ is the interaction term in which *E*_12_ is the epistatic effect size (user-defined, zero by default); α *_j_* is the effect size for *j ^th^* covariate *X*.

### Output files

Output files are in the formats as the direct inputs for the following tools: *EMMAX* ^14^, *Blossoc* ^4^, *Plink* (.ped file), *QTDT* ^15^, *TASSEL* ^16^, *GenABEL* ^17^ (see Table 1).

**Table 1.** Output file formats supported by phenotype simulator *cophesim*. Applying one of the options shown below controls the output format. Each output format has a special suffix type, which defines the file format. These output formats are concordant to those used in *phenosim*.

Application	Option	Commentary
EMMAX	-emmax	Suffices .emma_geno, .emma_pheno
BlOSSOC	-blossoc	Suffices .blossoc_pos, .blossoc_geno
PLINK	-plink	Used by default across all phenotypes, except survival. Suffices .ped, .map, .pheno.
QTDT	-qtdt	Suffices .ped, .map, .dat
TASSEL	-tassel	Suffices .poly, .trait
GenABEL	-	This format is used in simulation of survival phenotype.

### Operation

*cophesim* is freely available for download from the following link: https://bitbucket.org/izhbannikov/cophesim. Requirements: *Python* v2.7.10 and newer, *plinkio* v0.9.6, R v3.2.4 and newer, *Plink* v1.07, - in order to run the examples. The user manual is provided in a separate file “cophesim.pdf” located in the program directory and is also available as Supplementary File 1.

## Use case

Below we present an example that shows simulation of genetic data and then simulation of three different phenotypic traits. Other examples and installation instructions are provided at the program website and also in the user manual. Refer to the user manual for description of input parameters.



                    #-------------------------------------------Example begins---------------------------------------#
#Step 1: genetic data simulation:
plink --simulate-ncases 5000 --simulate-ncontrols 5000 --simulate wgas.sim --out sim.plink --make-bed
#Step 2: Convert .bed to .ped:
plink --bfile sim.plink --recode --out sim.plink
#Step3: phenotype simulation from previously made genetic data:
python cophesim.py -i sim.plink -o testout -itype plink -otype plink -c -ce effects.txt -s -gomp
#-------------------------------------------End of example---------------------------------------#
                


In this example, we first (Step 1) simulate genetic data using *Plink*. We simulate *N*. *cases* = *N*. *control* = 5,000 cases and controls and 1,000 SNPs (defined in wgas.sim file, refer to the *Plink* website to see documentation for this type of file). Then (Step 2) we convert a binary sim.plink.bed file to sim.plink.ped (option --recode in Plink). This step is not required since cophesim can handle binary *Plink* files ( .bed, .bim, .fam), but we provide this step in order to show the ability of the program to deal with *Plink* PED format. Finally (Step 3), we simulate dichotomous (by default), continuous (option -c) and survival (option -s) traits from previously simulated data stored in files sim.plink.ped and sim.plink.map. Note that we simulate survival trait with Gompertz hazard function (option - gomp); effect sizes for causal variants are provided in file effects.txt (to include this file we use option -ce).

### ROC curves

**Figure 2.**
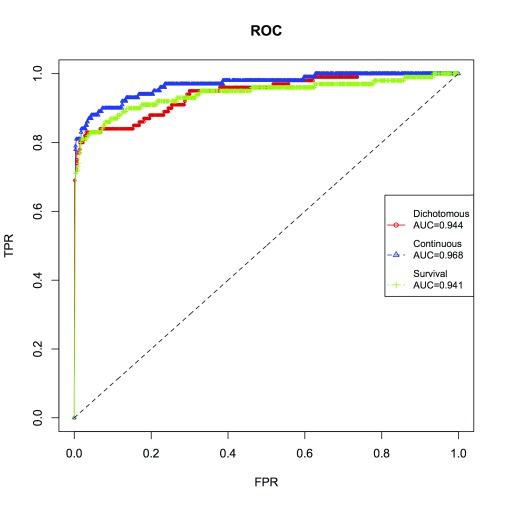
ROC curves constructed from results of association tests performed on a simulated dataset of *N* = 10,000 individuals, 100 causal and 1,000 of total SNP sites. TPR: True Positive Ratio, FPR: False Positive Ratio. These results were calculated for dichotomous, continuous and survival traits. The dashed, 45 degrees line represents random guessing.

We provide Receiver-Operating Characteristic (ROC) curves ( Figure 2) constructed from association tests performed on a simulated dataset. Simulation and association testing were performed with *Plink* suite. The following parameters were used: *N* = 10,000 individuals, *N*. *snp*. *c* = 100 causal, with total *N*. *snp* = 1,000 variants. Causal variants were labeled with ‘1’ and the other (neutral) variants were labeled with ‘0’. These labels are then used later as true identifiers during calculation of TPR (true positive rate) and FPR (false positive rate). Dichotomous, continuous and survival phenotypic traits were simulated with *cophesim*. Then association tests were performed with *Plink* for dichotomous and continuous traits (using *Plink* flags –logistic and -regression, respectively). Association tests for survival trait were performed with the R package *GenABEL*. Then calculated *p*-values provided by association tests for each variant were compared to the significance threshold. Those variants passed the threshold were recognized as causal and associated with simulated phenotype. These classification results later were compared to the true identifiers (defined above) in order to obtain TPR and FPR. For all these tests, we varied the significance threshold from 0 to 1 with the increment of 0.001.

The R code to construct ROC curves is provided in the file “roc.R”. This file is attached to this computer note and also in the data repository: https://bitbucket.org/izhbannikov/cophesim_data/ROC/roc.R

## Conclusion

In this work we presented the *cophesim* for phenotype simulation from genetic data obtained either from simulation or real data collecting. *cophesim* makes it possible to simulate various demographic models under user-defined scenarios.

## Software and data availability

Tool and source code available from: https://bitbucket.org/izhbannikov/cophesim

Archived source code as at time of publication: doi: 10.5281/zenodo.810195 ^18^

License: MIT

The example script and output files for the software are available at: https://doi.org/10.5281/zenodo.804090 ^19^.

To test the *cophesim* we provided a repository “cophesim_data”: https://bitbucket.org/izhbannikov/cophesim_data. Download or clone this repository to be able to run tests.

## Supplementary material

Table S1: Best available phenotype/genotype simulation software applications and their comparison to *cophesim* in terms of ability to simulate different types of phenotypic traits.

Supplementary File 1: User manual for *cophesim*.
