# Supplementary material for: cophesim: a comprehensive phenotype simulator for testing novel association methods
Source: F1000Res. 2017 Aug 1;6:1294. [Version 1] doi: 10.12688/f1000research.11968.1 (PMC5605948; doi:10.12688/f1000research.11968.1)
Supplement: Supplementary file 1 [file f1000research-6-12938-s0000.tgz › c65c7ddd-d305-4043-a722-e850f2413f10.docx]

Table S1. Best available phenotype/genotype simulation software applications and their comparison to *cophesim* in terms of ability to simulate different types of phenotypic traits. All of the presented applications (excluding phenosim and *cophesim*) are genotype simulators, which provide simulation of phenotypic traits as an additional feature. *cophesim* and phenosim are “pure” phenotype simulators without offering genetic simulation.

| **Application** | **Features** | | | | | | |
| --- | --- | --- | --- | --- | --- | --- | --- |
|  | **Dichotomous trait** | **Continuous trait** | **Survival trait** | **Gene-environment interactions** | **Epistatic interactions** | **LD simulation** | **Simulate phenotype from third-party genetic data**** |
| ***cophesim*** | **X*** | **X** | **X** | **X** | **X** | **X** | **X** |
| CoaSim | X |  |  |  |  |  |  |
| Fregene | X | X |  |  |  |  |  |
| ForSim | X | X |  |  |  |  |  |
| phenosim | X | X |  |  | X |  | X |
| QuantiNemo | X | X |  |  |  |  |  |
| PLINK | X |  |  |  |  |  |  |
| GCTA | X | X |  |  |  |  | X |
| GENOME | X | X |  |  |  |  |  |
| HapGen | X | X |  |  |  |  |  |
| SeqSimla | X | X |  |  |  |  |  |
| SimRare | X | X |  |  |  |  |  |

* Indicates ‘YES’

** When data was simulated with other tool or experimental data
