# Supplementary material for: cophesim: a comprehensive phenotype simulator for testing novel association methods
Source: F1000Res. 2017 Aug 1;6:1294. [Version 1] doi: 10.12688/f1000research.11968.1 (PMC5605948; doi:10.12688/f1000research.11968.1)
Supplement: Supplementary file 2 [file f1000research-6-12938-s0001.tgz › 42ab5de2-8130-4b8c-a7ce-abb2f3d55648.pdf]

December 17, 2016

cophesim
User Manual

Ilya Y. Zhbannikov[1], Konstantin G. Arbeev[1], Anatoliy I. Yashin[1]

[1]Biodemography of Aging Research Unit (BARU) at Social Sciences Research Institute (SSRI), Duke University, Durham, NC,

Keywords:
Bioinfomatics, Genomics, Data Simulation, Artificial Data, Synthetic Data

Correspondence:
Ilya Y. Zhbannikov
Biodemography of Aging Research Unit (BARU) at Social Sciences Research Institute (SSRI)
Duke University
Durham, NC, 27705
Email: ilya.zhbannikov@duke.edu

Running Title:
cophesim: a user manual

1 Introduction

cophesim - a comprehensive phenotype simulator for genetic data, i.e. cophesim adds phenotype to provided genotype files.

2 Installation

2.1 Prerequisites

- Python v2.7.10
- plinkio v0.9.6
- R v3.2.4 (for the examples)
- PLINK v1.7 (for the examples)

2.2 How to install

cophesim is an open-source software application available from the Bitbucket for free under this link: http://bitbucket.org/izhbannikov/cophesim.

1. Install prerequisites: Python, plinkio (if you were not able to install plinkio, just skip it. The “‘cophesim“‘ will still work, but not able to handle binary (.bed, .bim, .fam) PLINK files, only .ped and .map files will be handled), R, PLINK.
2. Download or clone the cophesim repository: https://bitbucket.org/izhbannikov/cophesim. Save under some name you wish and unzip. The software is ready to use.
3. Additionally, you may download the data repository: https://bitbucket.org/izhbannikov/cophesim_data. It provides some simulated data and code examples.

Save the file under some name you wish and unzip. The software is ready to use.

2.3 Usage

```
cophesim.py -i <path to genotype> -o <output prefix> [options]
```

2.4 Options

Input option described in Table 1

2.5 Description of input files

Input genotype data can be in one of the formats generated from the following applications: Plink (.bed, .bim, .fam); ms, msms, msHot (plain text file); Genome (plain text file). Plink format is used by default. In this case you have to provide a path to the files (i.e. full prefix without file extension).

2.6 Description of output

`cophesim` generates the following output files:

1. Phenotype file. This file is in text format and has the following suffices depending on the simulated phenotype trait: `_pheno_bin.txt`, `_pheno_cont.txt`, `_pheno_surv.txt` representing dichotomous (binary), quantitative (continuous) and survival phenotype. Below we show description of columns.

   `_pheno_bin.txt`:

   - Individual ID
   - Sex
   - Phenotype

Table 1: Input options

Option	Extended option	Description
-h	–help	Show the help message and exit.
-i IDATA	–input IDATA	Path input file(s). Extension should not be used in itype = plink.
-o OUTPUT_PREFIX	–output OUTPUT_PREFIX	Output prefix.
-itype ITYPE		Input format: `plink` (for Plink, default), `ms` (for ms, msms, msHot), `genome` (for Genome).
-otype OTYPE		Indicates output format, by default OTYPE=plink. Other possible output format: `blossoc` (for BLOSSOC), `qtdt` (for QTDT), `tassel` (for Tassel), `emmax` (for EMMAX).
-d	–dichotomous	A flag for dichotomous phenotype, True by default.
-c	–continuous	A flag for continous phenotype, False by default.
-s	–suvival	A flag to simulate survival phenotype, False by default.
-ce CEFF		A path to the file with effect of each causal SNP. Must be in format: `snp_index:effect`. One snp per line.
-alpha ALPHA		An 'alpha' parameter for inverse probability equation for the Gompertz hazard (see Bender at al., Generating survival times to simulate Cox proportional hazards models), 2005. Default ALPHA = 0.2138
-epi EPIFILE		File with interacting SNPs. One pair per line. Format: `snp1_index,snp2_index,effect`
-weib		A flag to use Weibull distribution for survival phenotype. True by default.
-gomp		A flag to use Gompertz distribution for survival phenotype. False by default.
-LD LDFILE		File with collinear SNPs. One pair per line. Format: `snp1_index,snp2_index,corr_cf([-1,1])`

`_pheno_cont.txt`:

- Individual ID
- Sex
- Phenotype

`_pheno_surv.txt`:

- Individual ID
- Sex
- Age (phenotype)
- Case

2. Genotype file(s). Can be in the following formats: Plink (`.bed`, `.bim`, `.fam`). Other possible output format: `blossoc` (for BLOSSOC, suffices `.blossoc_pos`, `.blossoc_geno`), `qtdt` (for QTDT, suffices `.ped`, `.map`, `.dat`), `tassel` (for Tassel, suffices `.poly`, `.trait`), `emmax` (for EMMAX, suffices `.emma_geno`, `.emma_pheno`).
3. Summary statistics file. This is a plain text file which keeps the information about the run.

2.7 Examples

Below we show several examples of usage of `cophesim`.

2.7.1 Quick start

```
plink --simulate-ncases 5000 --simulate-ncontrols 5000 --simulate wgas.sim \
--out sim.plink --make-bed

python cophesim.py -i sim.plink -o testout
```

The first command runs the data simulation. Here we simulate genetic dataset of 10k individuals, 5k cases and 5k controls. SNPs defined in `wgas.sim` (should be in the `cophesim` home directory). Then with next command we add a phenotype (dichotomous by default) to simulated genetic data.

To simulate continuous phenotypic trait, add the '-c' flag:

```
python cophesim.py -i sim.plink -o testout -c
```

This will simulate both continuous and dichotomous traits. To simulate survival trait, add '-s' flag:

```
python cophesim.py -i sim.plink -o testout -s
```

2.7.2 Specifying causal variants

Causal variants are specified in the file `effects.txt` and the option '-ce' is used:

```
python /Users/ilya/Projects/cophesim/cophesim.py -i sim.plink -o testout \
-ce effects.txt
```

The file effects.txt if a plain text file and causal SNPs are specified in the following format:

```
snp_index:effect
```

Here `snp_index` is the index of causal SNP and `effect` is the effect size. Example: `19:-0.82`.

2.8 Specifying epistatic interactions

Epistatic interaction are specified in the 'epifile.txt' with the '-epi' flag:

```
python /Users/ilya/Projects/cophesim/cophesim.py -i sim.plink -o testout \
-ce effects.txt -epi epifile.txt
```

The file epifile.txt if a plain text file and a pair of interacting SNPs is specified in the following format:

```
snp1_index,snp2_index,effect
```

Here `snp1_index` is the index of the first interacting SNP (snp1), `snp2_index` is the index of the second interacting SNP (snp2) and `effect` is the corresponding effect size of this interacting pair. Example: `12,16,1.57`.

3 Acknowledgements

This work was supported by the National Institute on Aging of the National Institutes of Health (NIA/NIH) under Award Numbers P01AG043352, R01AG046860, and P30AG034424. The content is solely the responsibility of the authors and does not necessarily represent the official views of the NIA/NIH.
