# Supplementary material for: cophesim: a comprehensive phenotype simulator for testing novel association methods
Source: F1000Res. 2017 Aug 1;6:1294. [Version 1] doi: 10.12688/f1000research.11968.1 (PMC5605948; doi:10.12688/f1000research.11968.1)
Supplement: Peer review history

# Peer review: Referee response for version 1

**DOI:** 10.5256/f1000research.12938.r25816

**Published:** 2017-09-18

**Authors:** Lars Rönnegård, Elena - Flavia Mouresan

**Affiliations:** 1 Dalarna University, Falun, Sweden; 2 Department of Animal Breeding and Genetics, Swedish University of Agricultural Sciences, Uppsala, Sweden

Zhbannikov and co-workers present the *cophesim* software that they have developed for simulating phenotypic data using genotype information. The input and output file formats are compatible with many of the most commonly used computer programs for genome wide association studies. The software is flexible, well documented and fills a gap in existing tools, especially for simulating time-to-event phenotypes. The paper is well written and easy to follow, and we only have some minor comments and suggestions.

 Minor comments

- Closing bracket missing in the sentence below equation (3)
- In equation (4), if the user does not provide gene effects then the phenotype is built by the sum of the standardized genotypes for each individual. Could you motivate this choice a bit and explain why it would be useful?
- In equation (5), the subscripts look wrong. a_i should be a_j
- In the Linkage Disequilibrium section the term “copula” is used. We do not think most readers of this paper can be expected to be acquainted with copulas and a reference is needed.

Consider adding a short paragraph where you discuss limitations and the possibility to add further functionality in the future, including:

- Dominance effects
- Probit link for binary data
- Simulation of correlated traits
- Alternative ways to simulate LD including a copula approach

 

 Check that the following link (at the end of the paper) works: https://bitbucket.org/izhbannikov/cophesim_data/ROC/roc.R (we were able to retrieve the code from https://bitbucket.org/izhbannikov/cophesim_data/src/)

We have read this submission. We believe that we have an appropriate level of expertise to confirm that it is of an acceptable scientific standard.

# Peer review: Referee response for version 1

**DOI:** 10.5256/f1000research.12938.r24718

**Published:** 2017-08-08

**Authors:** Arnold B. Mitnitski

**Affiliations:** 1 Department of Medicine, Dalhousie University, Halifax, NS, Canada

In the manuscript “Cophesim: a comprehensive phenotype simulator for testing novel association methods”, I. Zhbannikov and colleagues from the Duke University, presented a software that allowed to generate genotype data prepared with a genetic simulator for the use in the investigations of the genome wide association study (GWAS) tools. The rational for development of the software is clearly explained. The idea of the study is to use computer simulations to model data with specific assumptions. Similar simulators are known but all of them do not allow simulate survival. There are several other disadvantage with the existing simulators reviewed by the authors. 

 The description of the software is technically sound. The methods section is clearly presented. Dichotomous phenotype are simulated according to the logistic model with the covariates being genetic variants and covariates. Continuous phenotypes are simulated using the linear regression. Survival phenotype is modeled using the proportional hazards with inverse probability method.

 The details of the code, methods and analysis allow replication of the software and its use by the others. The methods section is clearly presented. Dichotomous phenotype are simulated according to the logistic model with the covariates being genetic variants and covariates. The output formats are compatible with the other applications (Table 1). It is useful example if using the simulator and the other examples are available in the manual. The ROC curve example is also very useful. The information provided is quite sufficient to allow interpretation of the expected results.

 In short, the Cophesim is a useful tool that can be helpful in the genetic analyses. The article is scientifically sound, the methods are described with details – this article will greatly help the researcher interested in the application genetic analyses.

I have read this submission. I believe that I have an appropriate level of expertise to confirm that it is of an acceptable scientific standard.
